# Anatomical Basis of Obstructive Sleep Apnoea: A Review of Randomized Controlled Trials

**DOI:** 10.7759/cureus.44525

**Published:** 2023-09-01

**Authors:** Shrikrishna B.H., Deepa G., Anupama Sawal, Trupti P Balwir

**Affiliations:** 1 Otorhinolaryngology - Head and Neck Surgery, All India Institute of Medical Sciences, Bibinagar, Hyderabad, IND; 2 Anatomy, Datta Meghe Medical College, Nagpur, IND; 3 Anatomy, Jawaharlal Nehru Medical College, Sawangi, IND

**Keywords:** hypoglossal nerve, tongue, mandible, tonsil, genioglossus, obstructive sleep apnoea

## Abstract

Repeated obstruction and closure of the upper airway, sporadic hypoxic episodes, and sympathetic activity are symptoms of obstructive sleep apnea (OSA). Obstructive sleep apnoea is due to a combination of altered upper airway structure and muscular function, a low arousal threshold and increased loop gain. Although recurrent upper airway (UA) collapse during sleep is the most frequent clinical hallmark of OSA, the exact cause of this collapse is unknown. Furthermore, while continuous positive airway pressure aids in the management of OSA, many patients find it intolerable. As a result, a better knowledge of the causes of OSA may result in more effective treatments.

We did a review of randomized controlled trials that were done in this regard in the last 10 years and whose full-text version is available on the PubMed database. A total of 20 articles were finalized for review after applying our criteria. The articles have proposed different theories regarding the anatomical basis responsible for obstructive sleep apnoea. The theories proposed by different studies in the last decade include reduced genioglossus and hypoglossal nerve activity, the pharyngeal muscles' failure to keep the airway open or tighten it, tonsils and adenoid hypertrophy, an oversensitive ventilatory control system and low respiratory arousal threshold, mandibular position, pharyngeal muscles' high sympathetic drive, cephalometric alterations such as mandibular and hyoid bone position and the length of the soft palate, obesity, and neck fat and fluid re-distribution in the body, from the lower to the upper parts while reclining.

Given the diverse etiological characteristics of OSA patients and to increase our knowledge of the condition, additional study into this group is required. Filling any knowledge gaps that may exist in the anatomical basis of the onset of OSA is the main objective of this review paper.

## Introduction and background

Intermittent hypoxic episodes are a hallmark of the widespread disorder obstructive sleep apnea (OSA) along with high sympathetic activity, together with repetitive collapse and closure of the upper airway [1]. The apnoea-hypopnea index (AHI), which measures the average number of apnoeas and hypopneas per hour of sleep, is typically used to describe the severity of the disease and can be classed as mild (AHI 5-15) grade, moderate (AHI 15-30) grade, or severe (AHI >30) grade [2-7]. The physiological characteristics of increased loop gain and a low arousal threshold, as well as altered upper airway structure and muscle function, combine to cause obstructive sleep apnoea (OSA). Untreated OSA raises the risk of several cardiovascular health issues, such as atrial fibrillation, congestive heart failure, stroke, hypertension, increased risk of car accidents, excessive daily sleepiness, a decline in quality of life, and a decline in social life. OSA has been linked to variations in the soft tissue and skeletal anatomy of the skull and face.

Although recurring upper airway (UA) collapse while sleeping is the primary clinical hallmark of OSA, the exact reason why this collapse occurs is not completely known. Additionally, even though continuous positive airway pressure helps treat OSA, many patients find it intolerable. So, a deeper comprehension of OSA causation might help with more effective therapies.

Given the various etiological traits of OSA patients, additional study of this cohort is required to increase our knowledge of the condition. Filling in any knowledge gaps regarding the anatomical causes of OSA development is the main objective of this review article.

## Review

Method

The main electronic databases that were searched were MEDLINE/PubMed and PubMed Central (PMC), which have the Medical Subject Headings (MeSH) keywords "Anatomy" and "Obstructive sleep apnea" in them. Our study includes studies with reports written in English, published within the last 10 years, and with full free text available on the database. Our search was not limited by age or gender. This review did not include studies that involved the use of animals or those that were written in a language other than English. The goal of our study was to identify the anatomical basis in the upper airway as an identifiable risk factor for obstructive sleep apnoea. The abstracts, titles, and free full-text versions of the publications were read by all of the writers of this review. The full text, abstract, publication date, title, name of the journal, and Digital Object Identifier (DOI) information for these articles were entered into Microsoft Excel sheets (Microsoft Corporation, Redmond, USA). Members of the team went over each item in the chart again to emphasize details in another chart that complemented our study objective. Because this was a standard literature review, no approval from the Research and Ethics Committee was required.

Result

For this review, a total of 20 studies were obtained. The 20 articles were all full-text and open-access as shown in Table 1.

**Table 1:** MeSH phrases were utilised to find pertinent studies.

Searches using MeSH (Medical Subject Headings) keywords- 'Anatomy' and ‘Obstructive Sleep Apnoea’	Count of records
Total records available	4558
Following the use of inclusion and exclusion criteria	
Randomized controlled trials	104
Published within the previous ten years	54
Free access to full texts	20

Discussion

The prevalent disorder known as obstructive sleep apnoea consists of repetitive narrowing and closure of the upper airway, as well as sporadic oxyhaemoglobin desaturation and sympathetic activity [1]. Daytime somnolence or sleep and a poor quality of life are consequences. It is a stand-alone risk factor for resistant hypertension. Vascular disease, insulin resistance, dyslipidemia, and mortality are all consequences of moderate-to-severe obstructive sleep apnoea, which is defined by an AHI score of 15 or more [2-7]. Lower drive to the upper-airway muscles is present at the onset of apnoea [8], and the genioglossus muscle is highly linked with upper-airway patency [9, 10].

In the study by Patrick J. Strollo et al., the upper-airway stimulation system was surgically implanted in qualified subjects. The tongue-protrusion function was activated by mounting the stimulation electrode on the hypoglossal nerve. The tongue muscles contract and the tongue moves anteriorly in response to electrical stimulation of the hypoglossal nerve. At a one-year follow-up, there were significant and clinically significant decreases in the severity of obstructive sleep apnoea and self-reported sleepiness as well as improvements in quality-of-life measures following unilateral stimulation of the genioglossal muscle or hypoglossal nerve in sync with ventilation [11]. By design, participants in this study had to have moderate to severe grades of obstructive sleep apnoea, experience a variety of continuous positive airway pressure (CPAP) compliance issues, and not exhibit complete concentric closure of the retropalatal airway during endoscopy in drug-induced sleep, or clinically severe central or mixed sleep apnoea. The obstructive sleep apnoea severity decreased in the cohort and the adverse event profile was tolerable.

Ursula G. Schulz et al. studied the association of OSA and leukoaraiosis and concluded that there is no relationship between obstructive sleep apnoea (OSA) and leukoaraiosis or age-related white matter changes. According to their research, leukoaraiosis is not strongly associated with OSA as an independent risk factor. In their cohort, the disorder was only mildly advanced. Their use of standard imaging sequences and assessment of apparent white matter alterations, which they believed would yield the clinically most reliable findings, may have been a limiting factor in their investigation [12].

Yan Shu et al. in their study opine that, although there is still debate over the precise pathophysiology of OSA in children, the majority of experts seem to concur with the idea that inhalation causes the patency of the upper airway to become constrictive or collapse. Such collapse is the end result of complex interactions comprising both functional and structural components. According to this hypothesis, it is crucial to remove the floppy airway in addition to the adenoid and tonsillar tissues to optimally widen the constriction and reduce the collapsibility of the pharyngeal area. They have recommended suturing the tonsillar wound to increase the retropalatal airways as much as possible and lessen the possibility of the lateral pharyngeal wall and soft palate collapsing [13]. Some otolaryngologists have gradually begun to suture the tonsillar pillars after tonsillectomy in infants with OSA. However, it is to be debated whether this tactic might have any negative effects, such as ones with postoperative velopharyngeal function.

Bradley A. Edwards et al., in their study, propose that for patients with obstructive sleep apnoea, oral appliances (OAs) are frequently utilised as a substitute for continuous positive airway pressure (CPAP). Upper-airway size is increased by OAs, which often reduces the severity of OSA and pharyngeal collapsibility. They propose that OSA is a complex illness that isn't just brought on by a dysfunctional upper-airway structure; several additional nonanatomic characteristics, such as (1) the airway not being held open or made rigid by the pharyngeal muscles, (2) a ventilatory control system that is too sensitive (high loop gain), and (3) a low threshold for respiratory arousal, also contribute to the pathogenesis of OSA. The mechanical stress on the airway may be reduced by enhancing the anatomy or collapsibility, which will increase the efficiency of the upper-airway dilator muscles in reopening the airway after airway collapse [14]. In this study, physiologic predictors of response to therapy were studied. In one aspect, the initial loop gain, which measures the ratio of how the body responds to changes in breathing and disruptions, was found to be important. People with lower initial loop gains experienced a better reduction in the severity of their obstructive sleep apnea when using an oral appliance. Additionally, there was a trend towards improvement in sleep apnea severity based on the airway's passive anatomy or collapsibility, meaning even if positive airway pressure is applied, the airway tends to collapse in some cases. The study's main flaw is that it only included 14 people, which is a rather small sample size.

In another study by Bradley A. Edwards et al., the authors propose that for individuals whose anatomy is not seriously impaired, an effective therapy for OSA involves reducing loop gain and raising the arousal threshold. The current study demonstrates that reduced loop gain and the ability to achieve lower breathing without arousal when oxygen is used in combination with eszopiclone treatment minimise the physiological gap that causes OSA. This combination of non-anatomical therapy improved the AHI in 95% of the patients examined. The combination of mandibular advancement and electrical genioglossus stimulation is effective in managing OSA, according to the authors [15]. Therefore, in a discernible fraction of carefully chosen patients, combining non-anatomical therapies can be successful in treating OSA.

Kazuomi Kario et al.'s analysis's key conclusion was that, when compared to the sham control group, the subset of OSA-resistant hypertensive patients receiving renal denervation demonstrated a higher decrease in office systolic blood pressure. In OSA patients utilizing continuous or bilevel positive airway pressure (CPAP/BiPAP), this difference in nocturnal blood pressure between renal denervation and sham control participants was also noted. The authors opine that patients with OSA frequently have episodes of hypoxia and hypercapnia during sleep, which operate through chemoreflex to stimulate a lot of sympathetic nerve activity. Upper airway blockage risk could be decreased by reducing sympathetic activity [16]. This study had its limitations in the sense that subjects self-reported having OSA, and baseline polysomnography wasn't done. However, this discovery is in concurrence with the discovery of Linz et al. who demonstrated in a preclinical model, that renal denervation, but not beta-blockade, reduced the enhanced sympathetic response linked to tracheal constriction [17].

According to Ying-Chun Cao et al., children with obstructive sleep apnoea have a morbidity rate of between 2% and 5%. For children with moderate to severe OSA and hypertrophied tonsils or adenoids, surgery is advised [18]. The study described has a two-centre randomised design, which significantly lowers the possibility of unfavourable impacts and selection biases. Every polysomnogram was assessed manually by researchers who were blind to the trial procedures that adhere to global norms. The trial's primary flaw is the small number of patients who were followed up with. According to Nieminen et al., OSA-induced growth hormone deficit is related to sleep patterns and sleep quality. Less slow-wave sleep and rapid eye movement (REM) occur in kids with OSA [19]. Hypoxia may prevent the hypothalamic-pituitary axis from properly regulating endocrine hormones, which could delay development [20].

According to Finn Geoghegan et al., regarding craniofacial measures, their study demonstrates that while the mandibular advancement devices (MAD) were in place in patients with OSA, the MADs altered several of the individuals' cephalometric traits. Significant improvements in face height, mandibular plane angle, overjet, soft palate length, and hyoid bone location with the mandible were also observed. The pharyngeal airway is controlled by the hyoid bone and its musculature in particular, and the location of the pharyngeal airway is influenced by the position of the tongue and the jaw [21]. In their study, the theory put out to account for the reduction in hyoid to mandibular plane distance is that the MAD positioned the mandible. This was a short-term study (6 months total). However, the length of this study was insufficient in order to represent the average life span of an OSA subject.

In a study by Blanca Ferrandez et al., when compared to healthy people, subjects with OSA demonstrated reduced sensitivity at the majority of white-on-white perimetry sites. In the periphery visual field, threshold values were lower. The authors think that retinal ganglion cells give a peripheral yet accessible window into central nervous system neurons, which may be destroyed as a result of OSA. They concluded that compared to healthy controls, OSA patients have lower retinal sensitivity as evaluated with standard automated perimetry [22]. The fact that the control group was not subjected to an overnight sleep study and it was presumed that they did not have any apnoea or hypopnea episodes per hour was a limitation of this investigation.

Talib et al. did a randomized controlled trial on 43 participants in 2020. The key conclusion from their research group of severely obese teenagers is that a higher left ventricular mass index is independently linked with male gender and higher apnoea-hypopnoea index, which suggests a pathophysiological connection between nocturnal hypoxemia and Left ventricular hypertrophy. They suggested that left ventricular hypertrophy risk factors include adiposity, which is unrelated to blood pressure and OSA is an important pathogenic factor in obesity-related left ventricular hypertrophy [23]. High methodological standardisation and thorough examination across numerous pertinent clinical domains are this study's main strengths. However, in this study, the sample size was small. Furthermore, since the majority of their cohort is female, it is impossible to verify that similar results apply to severely obese male teenagers. 

Jouett NP et al. opine through their study that intermittent hypoxemia, a feature of obstructive sleep apnoea, results in elevated sympathetic nerve activity. Their study professes that intermittent hypoxia training (IHT) causes significant increases in artery pressure and muscular sympathetic nerve activity that last even when people breathe normally in room air. This offers a potentially significant mechanistic explanation for the ongoing daytime muscle sympathetic nerve activity elevations that are seen in OSA patients [24]. The primary way in which this analysis is constrained is by not looking at a wider time window for the post-IHT recovery phase. Also, the recurrent respiratory episodes that many OSA patients experience over months and years are not replicated by this model of hyperacute intermittent hypoxia.

Schmickl CN et al. professed in their study that reduced pharyngeal muscle tone when sleeping is one of the primary causes underlying OSA. In OSA, the pharyngeal muscles' central activity decreases significantly and quickly during the wake-sleep transition, which may be a factor in the emergence of apnoeas and hypopnoeas. A decline in noradrenaline levels may also be important. A buildup of respiratory stimuli, such as an increase in intra-pharyngeal negative pressure and CO2, results in a progressive activation of the pharyngeal muscles following a mechanical collapse of the upper airway. The outcome of stable breathing depends on maintaining sleep until airway patency is restored; however, if the individual awakens before the airway opens, recurrent cycles of airway collapse and arousals (i.e. OSA) are predicted. The authors proposed that pharyngeal muscle tone can be elevated by pharmacologically enhancing central serotonergic/adrenergic tone [25]. However, this study is limited in the aspect that the authors have studied the effect of a single dose of venlafaxine on OSA. But it is difficult to predict the pharmacokinetic effects of a single 50-mg dose of venlafaxine for serotonergic/norepinephrinergic tones.

In osteopathic practice, the sphenopalatine ganglion's (SPG) intraoral myofascial massage is frequently utilised to treat snoring, chronic rhinitis, and nasal blockage [26]. This treatment likely enables people with temporo-mandibular joint dysfunction to achieve muscle relaxation and pain relief [27]. Jacq O et al. opine that OSA's aetiology may involve impaired upper airway function. The muscles that dilate the upper airways, especially genioglossus, which are mostly innervated by the hypoglossal nerve, are necessary to maintain their patency throughout the breathing cycle. The SPG, an autonomic nervous system ganglion, relays mixed cranial nerves from the upper airways. Osteopathic manipulative treatment of the SPG is used as an empirical therapy for rhinitis and snoring and is also thought to enhance pharyngeal stability in OSA patients [28]. This study suggests that, although the precise mechanism of action and duration of the effects cannot be identified, osteopathic manipulation of the SPG results in a neuromodulation effect and promotes pharyngeal stability. This study found a potential impact of osteopathic manipulation of the SPG on awake persons' upper airway stability despite the small sample size, but it does not support any conclusions about the effectiveness of this intervention in the management of OSA.

The relationship between craniofacial morphology and OSA is becoming more widely recognized [29], and it is more prominent in Asian populations than in Caucasian populations [30,31]. Goh KJ et al. [32] opine in their study that quantitative photography of the cranium appears to be able to predict the presence of OSA. In their study, they found that CPAP treatment for OSA patients resulted in improved adherence with nasal masks and worse adherence with oronasal masks. They hypothesised that the upper airway blockage is caused by pressure exerted through the mouth pushing the tongue and soft tissues backwards. Mouth breathers utilising oronasal masks as opposed to nasal masks have been reported to experience higher airflow blockage (less retropalatal airway opening) [33]. Another study employing real-time nasal endoscopy showed that while patients were wearing oronasal masks as opposed to nasal masks, the retroglossal area was substantially smaller and the distance between the epiglottis and tongue base was shorter [34]. These indicate that, despite nasal obstruction, nasal interfaces should still be considered when CPAP is started. However, this study has a shortcoming in that self-reported symptoms were used to quantify nasal obstruction, which may not be a good indicator of true nasal airflow resistance.

Carter SG et al. state in their study that OSA, as assessed by the AHI, is related to genioglossus movement patterns while silent breathing awake. In contrast to airway dilatation during sleep, it is hypothesised that increased genioglossus neural drive combined with counterproductive genioglossus motion during waking will induce velopharyngeal closure. The authors recommend that drugs such as zolpidem can have excitatory effects on the baseline activity of the genioglossus, the biggest dilator muscle in the upper airways [35]. This study had its limitations, including a small sample size, and even though sleeping supine was required for the participants, if individuals shifted to the lateral position during the night, the investigators did not intervene.

Doff MH et al. [36] opine that oral appliances that cause mandibular protrusion aid patients with OSA but they are viewed as a lifelong treatment option. However, oral appliances may cause dental problems like lessening of overjet and overbite, sub-optimal occlusion, and altered aesthetics. Blake KV et al. [37] opine that adenoids and tonsils play a pivotal role in causing OSA in children and adenotonsillectomy is the most common (90%) the operation is performed in children 0 to 6 years old for airway obstruction or sleep disruption.

In their study, Kasai T et al. opine that obesity-related indicators such as a higher body mass index (BMI) and circumference of the neck (NC) are significant obstructive sleep apnoea risk factors in the general populace. However, as measured by the sleep apnea and hypopnea frequency per hour, BMI and NC only contribute around 4% and 29%, respectively, to the variability in OSA severity. Upper airway (UA) obstruction in OSA patients must thus be caused by additional causes. One of these factors could be fluid transfer from the legs to the neck while sleeping on your back. An increase in the volume of neck fluid may lead to an increase in tissue pressure, which would constrict the UA and make it more likely to collapse. In conjunction with nuchal fluid build-up, fluid displacement from the legs while lying in bed may constrict the upper airway, which may assist in the aetiology of obstructive sleep apnoea. The upper airway lumen can become constricted and have an impact on the AHI when fluid from the legs is displaced into the soft tissue of the peri-pharynx while one sleeps. Therefore, in OSA patients, reclining may cause pharyngeal constriction and raise the likelihood of its collapse during sleep because of fluid transfer from the lower to the upper body. On the other hand, there seem to be mechanisms that cause UA dilation in response to the rostral fluid shift in people without sleep apnoea, which could prevent the onset of OSA [38].

Yadollahi et al. opine that the movement of fluid from the legs towards the neck during the night can contribute to the pathophysiology of OSA since individuals with fluid-retaining conditions like renal failure and heart failure have considerably higher rates of OSA than the general population. The substantially larger increase in AHI in response to saline infusion in older men compared to younger men may have been caused by the possibility that older people are more likely to have negative effects from fluid build-up in the neck because ageing alters the upper airway's physical characteristics. Age-related increases in upper airway collapsibility and resistance during sleep lend credence to this theory. Additionally, upper airway dilator reflexes may weaken with age, and older people (those over 40) have much weaker ventilatory and genioglossus muscle responses to hypoxia than younger people [39]. This study had its fallacies. Only non-obese subjects (BMI<30) were used in this study. The results might not be relevant to obese people as a result. Additionally, the study only included cases of non-severe OSA (AHI<30).

Glos M et al. in their study comparing MAD with CPAP opined that when it comes to preventing respiratory episodes, CPAP is superior to MAD. They also conclude that MAD's potential benefits in OSA will vary depending on aspects including younger age, lower weight, female gender, and certain craniofacial characteristics [40]. The absence of a washout time while switching from CPAP to MAD or MAD to CPAP is a limitation of this crossover trial.

Patrick J. Strollo et al., Bradley A. Edwards et al., Jacq O et al., and Carter SG et al., concur on the role of the reduced genioglossus and hypoglossal nerve activity as a contributor to OSA. The pharyngeal muscles are unable to maintain an open or rigid airway is the reason proposed by Shu Y et al., Bradley A. Edwards et al., and Schmickl CN et al. Tonsils and adenoid hypertrophy are suggested as a cause of OSA by Shu Y et al., Ying-Chun Cao et al., and Blake KV et al. The mandibular position is responsible in some cases of OSA which is helped by mandibular advancement devices according to Bradley A. Edwards et al., Finn Geoghegan et al., Doff MH et al., and Glos M et al. Pharyngeal muscles' high sympathetic drive is proposed as the aetiology for OSA by Kazuomi Kario et al. and Jouett NP al.

Cephalometric alterations such as mandibular and hyoid bone position and the soft palate's length contributes to the cause of OSA according to Finn Geoghegan et al. and Goh KJ et al. The theory of the relationship between obesity and OSA has been proposed by Ali Talib et al. Kasai T et al. and Yadollahi A et al. concur that fluid redistribution to the upper body from the lower while reclining may aid in pharyngeal narrowing and raise the chance of it collapsing as you sleep, more commonly in elderly people. According to Blanca Ferrandez et al., when compared to healthy persons, OSA sufferers have considerably lower retinal sensitivity. This appears more as an effect of OSA than a cause. The summary of the review can be seen in Table 2.

**Table 2:** Summary of the reviewed articles

Reference	Study Design	Year of Publication	Sample Size (n)	Finding
Patrick J. Strollo et al. [11]	Randomised control Trial	2014	126	Following unilateral stimulation of the genioglossal muscle or hypoglossal nerve in sync with ventilation, there were significant and clinically significant reductions in the severity of obstructive sleep apnoea and self-reported sleepiness as well as improvements in quality-of-life measures in patients with obstructive sleep apnoea who had difficulty accepting or adhering to continuous positive airway pressure (CPAP) therapy at one-year follow-up.
Ursula G. Schulz et al. [12]	Randomised controlled Trial	2013	183	There is no relationship between obstructive sleep apnoea (OSA) and leukoaraiosis or 'age-related white matter changes.
Shu Y et al. [13]	Randomised controlled Trial	2018	603	During inspiration, the patency of the upper airway is compromised or collapses. Such collapse is the the comprehensive outcome of several multifactorial interactions involving functional and anatomical components. The combination of pharyngoplasty and tonsillectomy offers considerable potential for treating children with obstructive sleep apnea.
Bradley A. Edwards et al. [14]	Randomised controlled Trial	2016	14	OSA is a complex illness that isn't just brought on by a dysfunctional upper-airway structure and several additional nonanatomic characteristics, such as (1) the inability of the pharyngeal muscles to keep the airway open or make it rigid, (2) a ventilatory control system that is too sensitive (i.e., high loop gain), and (3) Low respiratory arousal threshold is another factor in the development of OSA.
Bradley A. Edwards et al. [15]	Randomised controlled Trial	2016	20	The combination of mandibular advancement and electrical genioglossus stimulation is effective in managing OSA. In individuals whose anatomy is not seriously impaired, reducing loop gain and raising the arousal threshold together is an efficient treatment for OSA.
Kazuomi Kario et al. [16]	Randomised controlled Trial	2016	535	OSA patients who received renal denervation had significantly lower office systolic blood pressure at 6 months than sham control patients. Renal denervation significantly decreased overnight systolic blood pressure in non-dippers (persons with a fall of less than 10% in nocturnal arterial blood pressure), which further demonstrated that renal denervation had an impact on these patients' sympathetic drive.
Ying-Chun Cao et al. [18]	Randomised controlled Trial	2018	72	The use of low-temperature plasma to remove tonsils from individuals with oropharyngeal blockage and obstructive sleep apnoea has been proven to be both safe and effective.
Finn Geoghegan et al. [21]	Randomised Controlled Trial	2015	45	Significant cephalometric alterations caused by mandibular advancement devices (MAD) around the hyoid bone's position and the soft palate's length suggests that MADs may affect the position of the local muscles and enhance patency of upper airway.
Blanca Ferrandez et al. [22]	Randomised controlled Trial	2014	191	Retinal sensitivity was significantly reduced in OSA patients compared with healthy subjects. Retinal ganglion cells provide a peripheral window into the central nervous system neurons, which may be damaged as a result of OSA.
Ali Talib et al. [23]	Randomised controlled Trial	2020	43	The majority of severely obese teenagers have Left Ventricular Hypertrophy, and unfavourable geometrical changes, particularly increased Interventricular septal thickness, are independently linked to several frequently occurring nonhemodynamic risk factors, such as OSA, dyslipidaemia, and insulin resistance.
Jouett NPet al. [24]	Randomised controlled Trial	2016	9	Intermittent hypoxia training (IHT) causes large increases in muscle sympathetic nerve activity and arterial pressure that lasts even when people breathe normally in the room air. This offers a potentially significant mechanistic explanation for the ongoing daytime muscle sympathetic nerve activity elevations are seen in OSA patients.
Schmickl CN et al. [25]	Randomised controlled Trial	2020	20	In patients with OSA, the pharyngeal muscles' central activity decreases significantly and quickly during the wake-sleep transition, which may be a factor in the the emergence of apnoeas and hypopnoeas. A decline in noradrenaline levels may also be important.
Jacq O et al. [28]	Randomised controlled Trial	2017	10	The muscles that dilate the upper airways, especially genioglossus, which are mostly innervated by the hypoglossal nerve, is necessary to maintain their patency throughout the breathing cycle. Osteopathic treatment of the sphenopalatine ganglion may help those with obstructive sleep apnea syndrome maintain pharyngeal stability.
Goh KJ et al. [32]	randomized crossover trial	2018	85	The relationship between craniofacial morphology and OSA is becoming more widely recognized, and it is more prominent in Asian populations than in Caucasian populations. Adherence to nasal masks, as opposed to oronasal masks and nasal pillows were highest for obstructive sleep apnoea (OSA) patients receiving continuous positive airway pressure (CPAP) therapy. When starting CPAP, nasal masks should be the first interface used.
Carter SG et al. [35]	Randomised controlled Trial	2016	12	OSA, as assessed by the apnoea-hypopnea index (AHI), is related to the genioglossus movement patterns while silent breathing awake. In contrast to airway dilatation during sleep is hypothesised that increased 23 genioglossus neural drive combined with counterproductive genioglossus motion during waking will induce velopharyngeal closure.
Doff MH et al. [36]	Randomised controlled Trial	2013	103	Oral appliances that cause mandibular protrusion aid patients with OSA but they are viewed as a lifelong treatment option.
Blake KV et al. [37]	Randomised controlled Trial	2019	75	Adenoids and tonsils play a pivotal role in causing OSA in children and adenotonsillectomy is the most common (90%) operation performed in children 0 to 6 years old for airway obstruction or sleep disruption.
Kasai T et al. [38]	Randomised controlled Trial	2014	24	The Upper Airway lumen can become constricted and have an impact on the AHI when fluid from the legs are displaced into the soft the tissue of the pharynx while you sleep. Therefore, in OSA patients, fluid redistribution from the lower to the upper body while reclining may aid in upper airway narrowing and increasing the chance of it collapsing as you sleep. On the other hand, there seem to be mechanisms that cause UA dilation in response to the the rostral fluid shift in people without sleep apnoea, which could prevent the onset of OSA.
Yadollahi A et al. [39]	Randomised controlled Trial	2014	17	In comparison to younger men, older men are more vulnerable to the negative impacts of loading intravenous fluid on the severity of obstructive sleep apnoea. The collapsibility of the upper airways in response to loading intravenous fluid or age-related changes in the quantity of the fluid that builds up in the neck could be to blame for this. Additionally, the Upper Airway dilator reflexes may weaken with age, and older people (those over 40) have much weaker ventilatory and genioglossus muscle responses to hypoxia than younger people.
Glos M et al. [40]	Randomised controlled trial	2016	40	The potential benefits of OSA will vary trial depending on aspects including younger age, lower weight, female gender, and certain craniofacial characteristics.

Strengths and limitations

The papers included in this review were randomized clinical trials. Hence, this study bears a higher level of medical research evidence. However, meta-analysis studies were not included in our study. Another limitation of the study is the absence of non-English literary works and the use of a limited electronic database (PubMed and PMC).

## Conclusions

The theories proposed for the causation of OSA include reduced genioglossus and hypoglossal nerve activity and the incapacity of the pharyngeal muscles to keep the airway open or tighten it, tonsils and adenoid hypertrophy, a low respiratory arousal threshold and a ventilatory control system that is too sensitive, mandibular position, pharyngeal muscles' high sympathetic drive, cephalometric alterations such as mandibular and hyoid bone position and the length of the soft palate, obesity and neck fat, and fluid redistribution to the upper body from the lower while reclining. Given the diverse etiological characteristics of OSA patients, more research into this cohort is needed to advance our understanding of the disease. Greater knowledge of the aetiology of OSA might result in more effective therapies.
